# Anticancer, antioxidant, and antibacterial activity of chemically fingerprinted extract from *Cyclamen persicum* Mill.

**DOI:** 10.1038/s41598-024-58332-z

**Published:** 2024-04-11

**Authors:** Fuad Al-Rimawi, Mahmoud Khalid, Zaidoun Salah, Malak A. A. Zawahreh, Sulaiman Mohammed Alnasser, Shifaa O. Alshammari, Fadel Wedian, Shaik Karimulla, Abdulrahman Almutairi, Fadi Ibrahim B. Alanazi, Hamad Owijan Alanazi, Ghassab M. Al-Mazaideh, Hiba-Allah Nafidi, Ahmad Mohammad Salamatullah, Amare Bitew Mekonnen, Mohammed Bourhia

**Affiliations:** 1 Chemistry Department, Faculty of Science and Technology, Al-Quds University, P.O. Box 2002, Jerusalem, Palestine; 2 Molecular Genetics and Genetic Toxicology Program, Arab American University, Ramallah, Palestine; 3 Department of Pharmacology and Toxicology, College of Pharmacy, Qassim University, 51452 Qassim, Saudi Arabia; 4 Department of Biology, College of Science, University of Hafr Al Batin, P.O. Box 1803, Hafr Al Batin, Saudi Arabia; 5 Department of Chemistry, Faculty of Science, Yarmouk University, P.O. Box 560, Irbid, 22163 Jordan; 6 Department of Pharmacy Practice, University of Hafr Al Batin, Hafr Al Batin, Saudi Arabia; 7 Disaster and Emergency Services Department, Health Affairs Directorate, Hafr Al Batin, Kingdom of Saudi Arabia; 8 Department of Pharmaceutical Chemistry, College of Pharmacy, University of Hafr Al Batin, P.O. Box 1803, 31991 Hafr Al Batin, Saudi Arabia; 9 Department of Food Science, Faculty of Agricultural and Food Sciences, Laval University, 2325, Quebec City, QC G1V 0A6 Canada; 10 Department of Food Science and Nutrition, College of Food and Agricultural Sciences, King Saud University, 11, P.O. Box 2460, 11451 Riyadh, Saudi Arabia; 11 Department of Biology, Bahir Dar University, P.O. Box 79, Bahir Dar, Ethiopia; 12 Laboratory of Biotechnology and Natural Resources Valorization, Faculty of Sciences, Ibn Zohr University, 80060 Agadir, Morocco; 13 Al-Quds University, Al-Quds Bard College, Biology Program, Natural Sciences Division, Al-Quds, Palestine

**Keywords:** *Cyclamen persicum*, Antioxidant activity, Antimicrobial activity, Anticancer activity, Free radical scavenging activity, Drug discovery, Plant sciences

## Abstract

In the last few decades, researchers have thoroughly studied the use of plants in Palestine, one of them is *Cyclamen persicum* Mill. **(C. persicum**). *Cyclamen persicum* has been historically cultivated since the 1700s due to its tuber. The tuber is known to stimulate the nasal receptors, thus triggering the sensory neurons. *Cyclamen persicum* has anti-inflammatory effects, reduces cholesterol levels, treats diabetes, and inhibits tumor growth. In this respect, in-vitro examination of antibacterial and anticancer activities and antioxidative potency of *C. persicum* ethanolic extract were evaluated. The antioxidative potency of the extracted plant material was determined spectrophotometrically using the DPPH free radical scavenging method and the HPLC–PDA method to evaluate its total phenolic content (TPC) and total flavonoid content (TFC). The experimental results demonstrated limited antibacterial activity of *C. persicum* extract against both gram negative (*E. coli*) and gram positive (*Streptococcus* and *S. aureus*) bacterial strains, with the observed zones of inhibition measuring less than 8 mm. On the other hand, powerful activity against MCF7 breast cancer as well as HT29 colon cancer cell lines was obtained. The findings also revealed potent inhibition of free radicals and the presence of maximal levels of natural products such as phenolic compounds and flavonoids, which supportits biological activities and powerful ability to scavenge free radicals. HPLC results showed the presence of numerous flavonoid and phenolic compounds such as rutin, chlorogenic acid, kaempferol, trans-cinnamic acid, quercetin, sinapic acid, and p-coumaric acid.

## Introduction

Throughout history, the utilization of plants has been used for medicinal purposes. The oldest records of using plants for medicinal purposes date back almost 5000 years and refer to over 250 different types of plants. Some plants that were recorded thousands of years ago, such as poppy (pain relief), henbane (sedative), and mandrake (an ingredient in making medicine), are still used today for their medicinal properties^1^. Palestine, with its unique geographical location and rich biodiversity, is known for its medicinal plants, with an estimate 2953 plant species^2^. Currently, more than10% of plants are used for their medicinal properties and contain alkaloids and terpenoids^3^. However, due to limited resources and studies conducted, much of the published data does not accurately reflect the actual status of medicinal plants in Palestine or the Palestinian territory^4,5^.

The study of the anticancer properties of plants dates back centuries. However, instead of a couple hundred anticancer medicinal plants, approximately 35,000 plant species are being studied for their anticancer properties, and 3000 plants have been shown to exhibit consistent anti-cancer effectiveness^6^. Plant-derived drugs are preferred for anticancer treatment as they are highly efficient and generally well-tolerated by the body, being non-toxic to human cells. However, with people turning away from chemotherapy due to its risky side effects, there is a significant demand for herbal medicine, leading to an increase in the diversity of medicinal plants^7–10^. Additionally, many medicinal plants have been found to be more efficacious in curing various infectious diseases and alleviating the side effects of synthetic antimicrobial drugs^11–15^.

**Figure 1:**
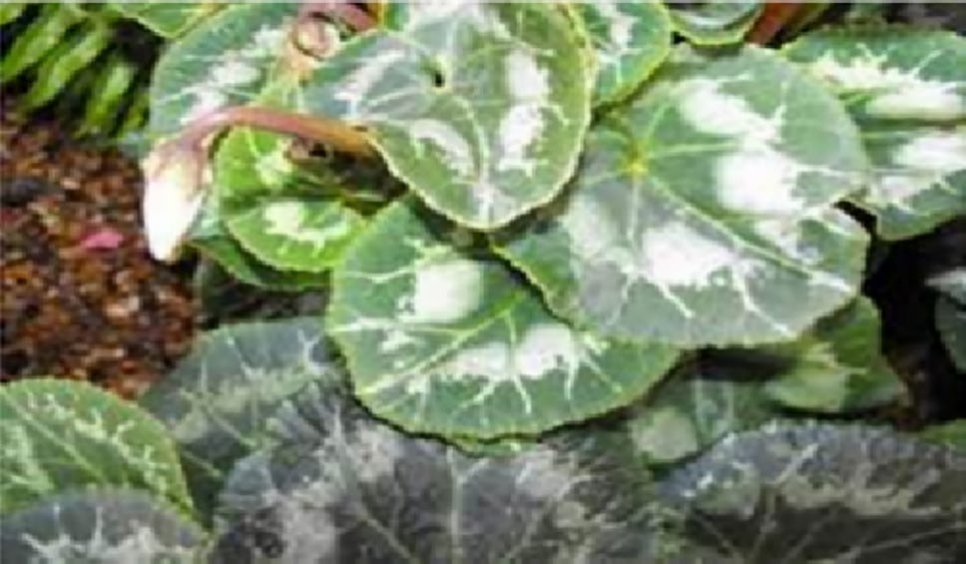
*Cyclamen persicum* Mill*.*

Within the Mediterranean area, and more specifically in Palestine, a widely used plant is *C. persicum* (Fig. 1). The Cyclamen plant has been used as a medicinal plant for several hundred years, particularly since the nineteenth century^16,17^. In traditional medicine in Palestine, *C. persicum* is regularly used to extract its root. Studies have shown that the root of *C. persicum* is rich in saponins, which are known to reduce cholesterol levels, treat diabetes, and inhibit tumor growth. It has been observed that the root is absorbed through the nasal mucosa but not into the bloodstream^18^.

The *C. persicum* flower is indeed extremely common in the Mediterranean region and has been cultivated since the 1700s^19^. It is recognized by its heart-shaped leaves and tubers, which are the primary reasons for its cultivation . The plant is considered semi-poisonous due to the presence of saponins in its tuber, which can stimulate nasal receptors and result in sensory or pain perception. However, studies have shown that when the saponin of *C. persicum*, particularly repanoside, is isolated, it can influence the response of human macrophages, thereby mediating inflammatory effects^20^.

The investigation was focused on *C. persicum* and its antimicrobial and anticancer properties. The objectives of the study are therefore to investigate the antimicrobial, anti-cancer, and antioxidant activities of the ethanolic extract of *C. persicum.* Additionally, the study aims to determine the polyphenolic and flavonoid of the extract, as well as identify the specific polyphenolic compounds present, using the HPLC–PDA method.

## Methodology

### Plant extraction

*Cyclamen* *persicum* plant samples were collected from Ramallah, West Bank/Palestine and then identified by Dr. Nidal Jaradat. A herbarium voucher number (Pharm-PCT-260) was assigned to the plant, and it was deposited in the herbarium of An-Najah National University The aerial parts of the plant were dried in the shade until a constant weight was achieved, and then they were pulverized. 10 g of the powdered material were separately soaked in 100 mL of ethanol (100% and 50%) using ultrasonic treatment for 3 h. The mixture was then filtered, and the solvent was concentrated using a rotary evaporator at a temperature of 47 °C). The resulting residue was dissolved in methanol to obtain a concentration of 0.1 g/mL.

### Antimicrobial testing

In order to assess the antimicrobial activity of the *C. persicum* extract, two gram-positive strains (*S. aureus* and *Streptococcus*) and one gram-negative strain (*E. coli*) were used. To create the Mullen-Hinton agar media, 19 g of agar media were combined with 500 mL of distilled water. The mixture was then autoclaved at 121 °C for 15 min. After autoclaving , the Mullen-Hinton agar was allowed to cool and poured into 6 culture Petri dishes. To culture *Streptococcus*, *S. aureus*, and *E. coli* on the petri dishes, pure colonies of each strain were obtained and inoculated into small vials containing distilled water. The McFarland turbidity standard was followed to prepare the inoculum. Using an inoculating loop, samples of inoculum were swabbed and streaked on the surface of the agar media in the 6 petri dishes. To create wells, a 100 µL pipette tip was inserted into the agar media, and the contents of the formed wells were removed to make circular holes. The *C. persicum* extract was then pipetted into the wells. The Petri dishes were incubated for 24 h at 37 °C to allow for microbial growth and the assessment of antimicrobial activity.

### Anti-cancer testing

To assess the cytotoxic activity of the *C. persicum* extracts, two cancer cell lines were used: HT29 colon cancer and MCF7 breast cancer cell lines, obtained from the American Type Culture Collection (ATCC), USA. Prior to the assessment, both cell lines were harvested and suspended in RPMI medium. The cells were then incubated for 24 h to allow them to settle and adapt to the culture conditions. For each cancer cell line, five tissue culture plates were prepared. The plates were divided into three parts in a ratio of 2:2:1. The first two parts were allocated for 100% and 50% ethanolic extracts of *C.* *persicum*, respectively, while the third part served as a negative control using DMSO (Dimethyl Sulfoxide). DMSO-diluted extracts were prepared at volumes of 100 μL and 50 μL, and they were added to the tissue culture plates independently for each cell line in the respective volumes. The tissue culture plates of each cell line were treated with 3 μL of 100% and 50% of DMSO diluted ethanol extracts and then incubated at 37 °C for 72 h.

### Activity of DPPH in scavenging free radicals

The DPPH antioxidant assay evaluates whether a test sample can scavenge or neutralize the free radical property of the stable DPPH radical. First, a DPPH solution of 0.062 mM was prepared using 95% methanol as the solvent. Later, a 3.9 mL portion of this solution was added to 0.1 mL of *C. persicum*, and the mixture was vortexed for 10 s and incubated at room temperature for 30 min. The absorbance of the DPPH solution was measured at 515 nm using 95% methanol as blank sample. A calibration curve was measured using different Trolox concentrations ranging from 20 to 200 ppm. The results were expressed as µmol Trolox per gram (µmol Trolox/g).

### Determination of Total Phenolic Content (TPC)

The total content of phenolic compounds in *C. persicum* plant extracts was determined using the Folin–Ciocalteu colorimetric method. The Folin–Ciocalteu reagent was diluted tenfold with distilled water, and 1.8 mL of this diluted reagent was added to a measured volume of the plant extract. The mixture was set aside for five minutes before adding 1.2 mL of the 7.5% NaHCO_3_. After 60 min at room temperature, the absorbance of the samples was recorded at 765 nm. The same procedure was repeated for the gallic acid standard. A calibration curve was constructed using aqueous solutions with known gallic acid concentrations ranging from 20 to 500 mg/L. The results were expressed in terms of gallic acid equivalents (GAE) per gram of the sample^21^.

### Determination of the total flavonoids content (TFC)

The total content of flavonoids in *C. persicum* extract was determined spectrophotometrically using the Aluminum chloride method. The following steps were followed using a test tube: 1 mL of *C. persicum* extract was mixed with 5 mL of distilled water, 0.3 mL of 5% sodium nitrate solution, and 0.3 mL of 10% aluminum chloride solution.The mixture was allowed to stand at room temperature for 5 min. Then 2 mL of 1M sodium hydroxide was added, and the final volume was adjusted to 10 mL using water. After the last step, the final solution was vortexed, and absorbance was measured spectrometrically at 510 nm. A standard calibration curve was prepared using different concentrations of catechin ranging from 20 to 100 mg/L. The results were expressed as milligrams of catechin equivalents (CEQ) per gram of the sample^21^.

### Phytochemicals HPLC analysis

#### RP-HPLC conditions

The bioactive compounds were evaluated using a Waters Alliance e2695 HPLC system connected to a 2998 PDA detector. Data acquisition and analysis were performed using Empower-3 chromatography information software. The reversed-phase HPLC method was employed to analyze various polyphenolic compounds as well as flavonoids. A C18 column with a length of 25 cm and an inner diameter of 3.6 µm was used. The mobile phase consisted of a combination of 0.5% solution A (CH_3_COOH-Acetic acid) and solution B (CH_3_CN-Acetonitrile), following a linear mobile phase configuration as outlined in Table 1. The flow rate was set at 0.5 mL/min, and the column temperature was maintained at approximately 25 ºC. An injection volume of 20 µL was used for the samples, which were filtered through a 0.45 µm filter prior to injection. During the analysis, a photodiode array (PDA) sensor with a wavelength range of 210–400 nm was utilized to detect and measure the compounds of interest.

**Table 1:** Conditions of the gradient mobile phase.

Time (min)	0.5% Acetic acid solution	0.5% Acetonitrile solution
0.0	95%	5%
50.0	80%	20%
65.0	65%	35%
70.0	40%	60%
75.0	10%	90%
78.0	95%	5%
80.0	95%	5%

#### Preparation of standard solutions

The following standards were used during the HPLC analysis:rutin, vanillic acid, ferulic acid, isovanillic acid, kaempferol, quercetin, verbacoside, p-coumaric acid, chlorogenic acid, sinapic acid, gallic acid, trans-cinnamic acid, caffeic acid, syringic acid, 3,4-dihydroxybenzoic acid, and 3,4-dihydroxyphenylacetic acid.. To prepare the standards mixture, 5 mg of each compound was dissolved in 5 mL of 20% ethanol. Distilled water was then added to create a volume of 25 mL. The standards mixture was injected (20 µL) into the HPLC chromatograph and analyzed using the aforementioned reversed-phase (RP) technique.

#### Preparation of plant extract samples for HPLC

A dry extract weighing exactly 100 mg was dissolved in 20 mL of 95% ethanol. Subsequently, 100 mL of distilled water was added to the solution.

### Statistical analysis

All experimental data analyses were performed using Microsoft Excel 2010. The reported results were presented as means ± standard deviation (SD). Statistical analyses were conducted using SAS (SAS Institute Inc., Cary, USA, Release 8.02, 2001).

To assess the impact of the extraction solvent on TPC, TFC, and AA, comparisons of means were performed using the General Linear Model (GLM) procedure. The main factor, which is the extraction solvent, was treated separately using one-way analysis of variance (ANOVA).

### Plant collection approval

No approval is needed from the authority in Palestine to collect *Cyclamen persicum* for research purposes. However, landowner approved the collection of *Cyclamen persicum* for research purposes.

### IUCN Policy statement

The collection of plant material complies with relevant institutional, national, and international guidelines and legislation.

## Results

### Total phenolic content (TPC)

The total phenolic content (TPC) was assessed by Folin Ciocalteu method, and the results were presented as milligrams of gallic acid equivalents (GAE) per gram of sample with reference to a calibration curve. The total phenolic content of the ethanolic extracts is presented in Table 2. For the extract with a 50% ethanol concentration, TPC was found to be 114.3 ± 2.2 mg of gallic acid/g. In contrast, the extract with 100% ethanol concentration contained a total of 102.4 ± 3.1 mg of gallic acid/g phenolics. The results demonstrated that the 50% ethanol extract exhibited greater extracting power for total phenolic compounds compared to the 100% ethanol extract. Statistical analysis confirmed this finding, as indicated by the capital letters A and B in Table 2, indicating that TPC of the 50% ethanolic extract was statistically higher than that of the 100% ethanol extract. The higher levels of polyphenolic substances observed in the 50% ethanol mixed mode, which contains both ethanol and water, may be responsible for this behavior. This difference can be attributed to the polarity of the solvent and its ability to saturate active phytoconstituents in plants.

**Table 2:** Antioxidant activity (AA), total phenolic content (TPC), and total flavonoid content (TFC) of *C. persicum.*

AA (µmol Trolox/g)		TPC (mg gallic acid/g)		TFC (mg catechin/g)	
Ethanol		Ethanol		Ethanol	
50%	100%	50%	100%	50%	100%
342.9 ± 4.9 A	210.7 ± 7.3 B	114.3 ± 2.2 A	102.4 ± 3.1 B	32.4 ± 2.5 A	22.3 ± 2.4 B

A and B indicates significant difference in AA, TPC and TFC using two extraction solvents, where A indicates statistically higher than B.

### Total flavonoid content (TFC)

The TFC was measured using Aluminium chloride method and expressed as milligrams of catechin per gram (mg catechin/g) with reference to a standard curve of catechin. The results for 50% and 100% ethanolic extract showed that the TFC was found to be 32.4 ± 2.5 and 22.3 ± 2.4 mg catechin/g, respectively. The TFC was observed to be higher in the 50% ethanolic and lower in the 100% ethanol extract. This difference can be attributed to the polarity of the solvent and the solubility of flavonoids in polar solvents. The higher polarity of the 50% ethanolic solvent may have facilitated better extraction of flavonoids, resulting in a higher TFC compared to the 100% ethanol solvent. These findings provide support for the ethanolic solvent extract of *C. persicum* having a high phenolic and flavonoid content. Statistical analysis confirmed that TFC of 50% ethanolic extract was statistically higher than that using the 100% ethanol extract).

#### Antioxidant activity (AA)

The AA of the extracts was assessed using the DPPH free radical test which utilizes a stable free radical. The results were reported in terms of micromoles of Trolox per gram (µmol Trolox/g). The findings indicated that the AA of the 50% ethanolic extract was measured at 342.9 ± 4.9 µmol Trolox/g, while the 100% ethanolic extract exhibited a lower AA of 210.7 ± 7.3 µmol Trolox/g. This variation in AA between the two extracts can potentially be attributed to the polarity of the solvents used. This variation in antioxidant activity between the two extracts can potentially be attributed to the polarity of the solvents used. The 50% ethanolic solution, which combines water and ethanol, was more effective in extracting flavonoids and phenolic compounds, known contributors to AA. This trend was consistent with the findings of the total phenolic and total flavonoid content, where the 50% ethanolic solvent demonstrated superior extraction of these compounds compared to the 100% ethanolic solvent.

### HPLC examination of flavonoids and polyphenolic compounds

**Figure 2:**
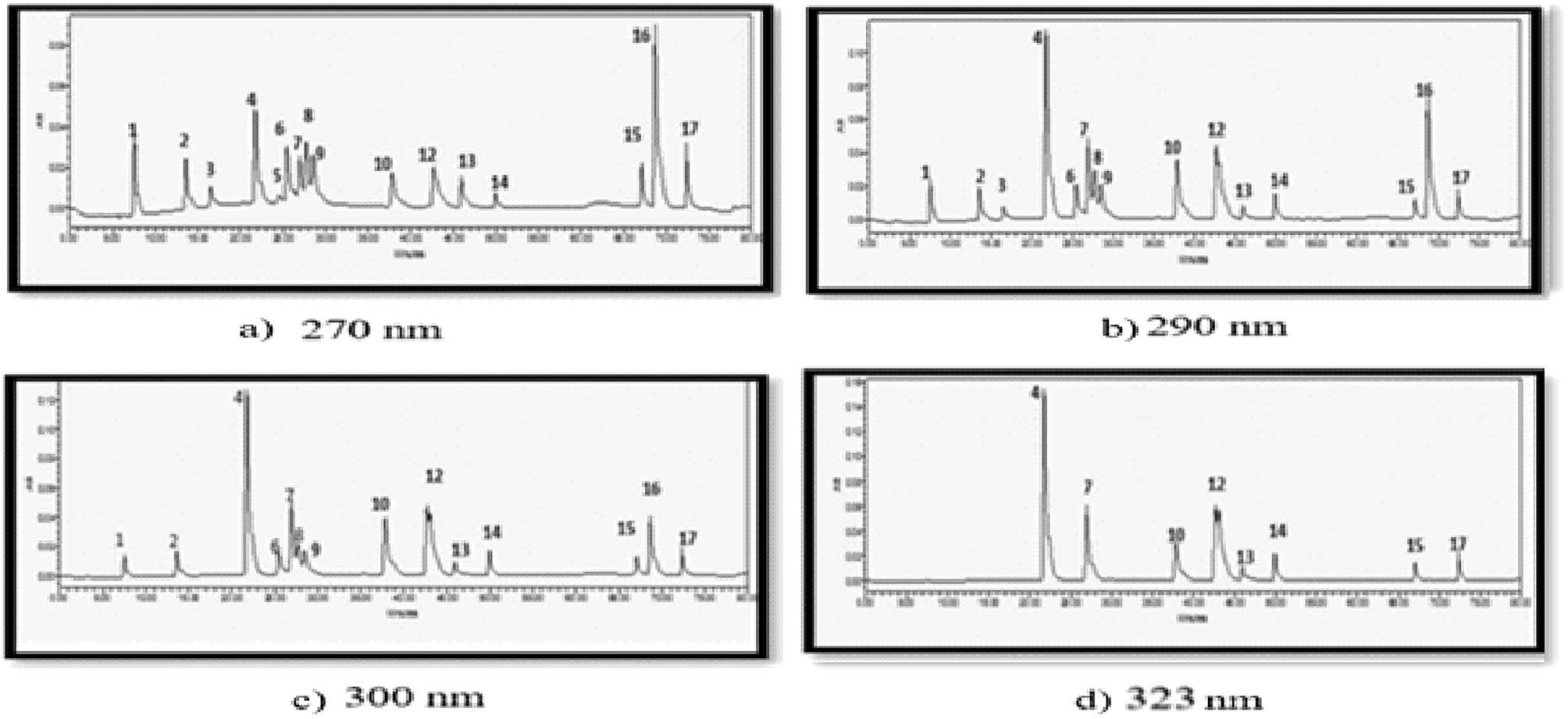
HPLC chromatogram of standards for polyphenols and flavonoids that were examined using the RP-HPLC method with different range of wavelengths (270 nm (**a**), 290 nm (**b**), 300 nm (**c**), and 323 nm (**d**)).

Twenty microliters (20 µL) of the mixture containing the 17 standards were injected into the HPLC chromatograph, following the previously mentioned RP-phase analysis method. A photodiode array detector was used, allowing for detection at multiple wavelengths since each substance has a unique wavelength with maximum absorption (as indicated in Table 3). The chromatograms of the standards mixture were obtained at different wavelengths, specifically 270 nm (a), 290 nm (b), 300 nm (c), and 323 nm (d), as shown in Fig. 2a–d. By using different wavelengths, the 17 compounds were successfully separated, as depicted in Fig. 2a–d. Table 3 provides a list of the retention times for each standard, along with the corresponding peak absorption wavelength for each compound.

**Table 3:** HPLC analysis results for the standard compounds analyzed in this study.

Phytochemical name	Rt (min)	Wavelength (nm)
Rutin	45.98	254
3,4-Dihydroxybenzoic acid	13.86	258
Isovanallic acid	28.54	258
Vanallic acid	25.41	261
Kaempferol`	72.35	264
Gallic acid	08.25	270
Syringic acid	27.72	273
4-hydroxyphenylacetic acid	24.54	273
Trans-cinnamic acid	68.68	274
3,4-Dihydroxyphenylacetic acid	16.56	281
p-Coumaric acid	37.81	308
Caffeic acid	26.91	321
Ferrulic acid	42.67	321
Chlorogenic acid	21.63	322
Sinapic acid	43.11	322
Verbascoside	49.99	328
Quercetin	67.03	363

**Figure 3:**
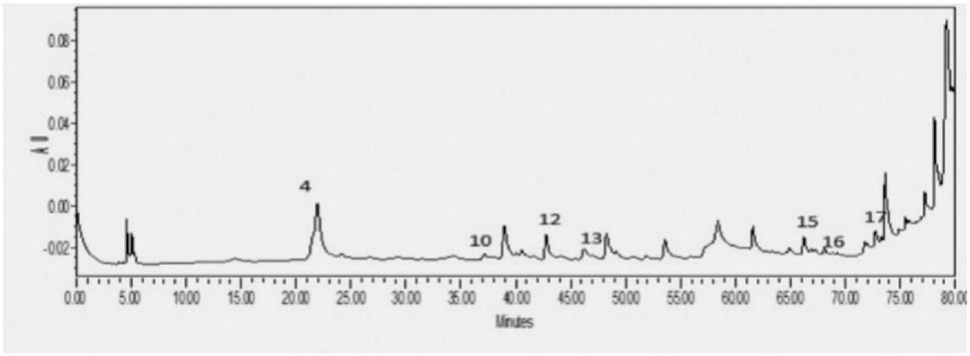
HPLC chromatogram of *C. persicum* at 300 nm.

Figure 3 displays the RP-HPLC chromatograms of *C.* *persicum* analyzed at a wavelength of 300 nm.This wavelength was selected because it showed the highest absorption for the major peaks. The eluted compounds were identified within the elution time range of 21 to 77 min, indicating the presence of both polar and nonpolar substances. Variouscompounds were identified in this extract, including rutin, chlorogenic acid, kaempferol, trans-cinnamic acid, quercetin, sinapic acid, and p-coumaric acid.

## Discussion

Comparing the TPC of *C. persicum* extract with other plant extracts or established standards can provide valuable insights into its potential health-promoting effects. A higher phenolic content is often associated with increased antioxidant activity, which is beneficial for human health. Phenolic compounds have been widely studied for their various biological activities, including antibacterial, anti-inflammatory, and anticancer effects. By understanding the TPC of *C. persicum*, we can further explore its potential therapeutic benefits and identify it as a potential source of valuable bioactive compounds.

Flavonoids, a subgroup of phenolic compounds, are renowned for their antioxidant and anti-inflammatory properties. Measuring the TPC in *C. persicum* extract provides valuable information about the presence of these beneficial compounds. Flavonoids have been associated with a range of health benefits, including promoting cardiovascular health, modulating the immune system, and exerting neuroprotective effects. Analyzing the flavonoid content helps in evaluating the potential health implications of consuming or utilizing *C. persicum*^22^.

Examining the correlation between the TPC and TFC can unveil the diversity of bioactive compounds present in the extract, highlighting the multifaceted antioxidant potential of *C. persicum*. A positive correlation between AA and the content of total phenolics/flavonoids validates the crucial role of these compounds in the observed antioxidant effects. The synergistic interactions among different bioactive components contribute to the enhanced antioxidant capacity of *C. persicum* extract.

In a study conducted by Murat Turan et al.^22^, the antioxidant and anti-proliferative activities of acetone, methanol, and water extracts from different parts (fresh and underground parts) of Cyclamen were investigated. The study determined the TCP and TFC and tannin contents in the extracts. The maximum values obtained were 3.69 ± 0.13 mg GAE/g-extract for TPC, 18.48 ± 0.12 mg QE/g-extract for TFC, and 41.17 ± 0.44 mg CE/g-extract for tannins. Furthermore, the extracts obtained from *C. persicum* displayed anti-proliferative activity in Caco-2 colon cancer cells. This suggests that the extracts from *C.* *persicum* may have potential therapeutic effects against colon cancer.

In a study by Ayadin et al.^21^, the phenolic composition and biological activities of various extracts of *C. persicum* were evaluated. The highest phenolic compound identified in the extracts was cinnamic acid, with a concentration of 411.6 µg/g. The ethanolic extracts demonstrated the highest TPC, measuring 8.99 ± 3.07 mg GAE/mL. Similarly, the ethanolic extracts also exhibited the highest TFC, with a value of 54.66 ± 3.02 mg QE/g. The radical scavenging activity of the extracts varied among the different solvents used for extraction. The tuber acetone extract displayed the highest radical scavenging activity, measuring 336.3 ± 0.02. On the other hand, the tuber ethanol extract showed a lower radical scavenging activity of 129.74 ± 0.02. Moreover, the tuber methanolic extracts exhibited strong inhibition of the growth of Gram-positive *S. aureus*. The minimum inhibitory concentration (MIC) for the tuber methanolic extract was determined to be 56 ± 0.03 μg/mL, indicating its significant potential as an antibacterial agent against *S. aureus*.

### Antioxidant activity (AA)

Based on Table 2, the AA, measured as DPPH free radical scavenging, was higher in the 50% ethanolic extract compared to the 100% ethanolic extract. The values of 342.9 ± 4.9 µmol Trolox/g for the 50% ethanolic extract and 210.7 ± 7.3 µmol Trolox/g for the 100% ethanolic extract. The presence of polyphenolic substances or flavonoids in the extracts may explain the observed AA. Flavonoids and polyphenolic compounds are well-known for their effectiveness as free radical scavengers, contributing to their antioxidant potential These compounds act as reducing agents, hydrogen donors, and singlet oxygen quenchers, collectively enhancing their antioxidant properties.

### Antimicrobial activity

The antibacterial activity of the plant extracts was evaluated using the disk diffusion method against three bacterial strains: *S. aureus* and *Streptococcus* (both Gram-positive bacteria), and *E. coli* (a Gram-negative bacterium). The results indicated that both the 100% ethanol extract and the 50% ethanol extract displayed weak antibacterial activity against all tested bacteria. The zone of inhibition, which indicates the area of bacterial growth inhibition around the disk containing the extract, was less than 8 mm for all bacterial strains, indicating low inhibitory effects.

### Anti-cancer activity

In the evaluation of the anti-cancer activity of *C. persicum*, the effects of the extract were tested on two cancer cell lines: MCF7, representing breast cancer, and HT29, representing colon cancer. The negative control used in the experiment was DMSO (dimethyl sulfoxide), which did not show any significant effect on the cancer cells, as indicated in Fig. 2a. This suggests that the observed effects on the cancer cell lines can be attributed to the *C. persicum* extract rather than the DMSO control.

### MCF7 breast cancer cell line

**Figure 4:**
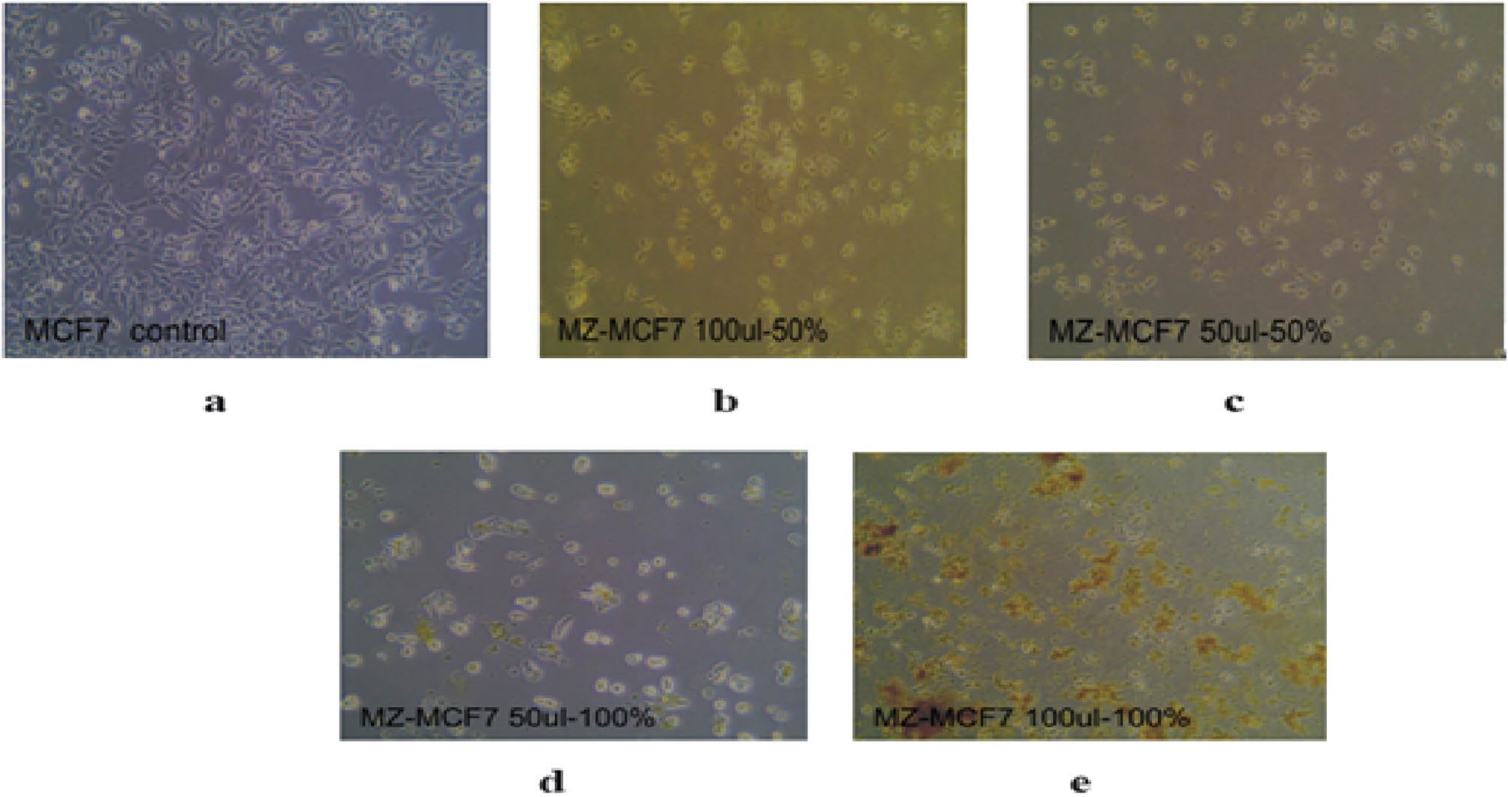
The effect of *C. persicum* on MCF7: (**a**). The control samples of the MCF7 cell lines 48 h after culturing with DMSO (**b**). MCF7 cell lines 48 h after culturing with 100 μL of 50% extract (**c**). MCF7 cell lines 48 h after culturing with 50 μL of 50% extract (**d**). MCF7 cell lines 48 h after culturing with 100 μL of 100% extract (**e**). MCF7 cell lines 48 h after culturing with 50 μL of 100% extract.

The results of the study demonstrated that *C. persicum* extract exhibited significant anti-cancer activity specifically against the MCF7 breast cancer cell line. The assessment involved cell counting and calculation of the percentage of cell inhibition, which indicated a noticeable decrease in cell count when treated with the plant extract compared to the negative control (DMSO). Comparing the 50% and 100% ethanolic extracts to the negative control, a clear decline in the number of cancer cells was observed. The anti-cancer activity was more pronounced at higher concentrations (100 µL) compared to lower concentrations (50 µL). Importantly, there was no significant difference in the activity against the MCF7 cell line between the 100% and 50% ethanolic extracts, as demonstrated in Fig. 4a–e of the study. This suggests that both concentrations of the ethanolic extract have similar efficacy in inhibiting the growth of MCF7 breast cancer cells.

### Colon cancer cell line (HT29)

**Figure 5:**
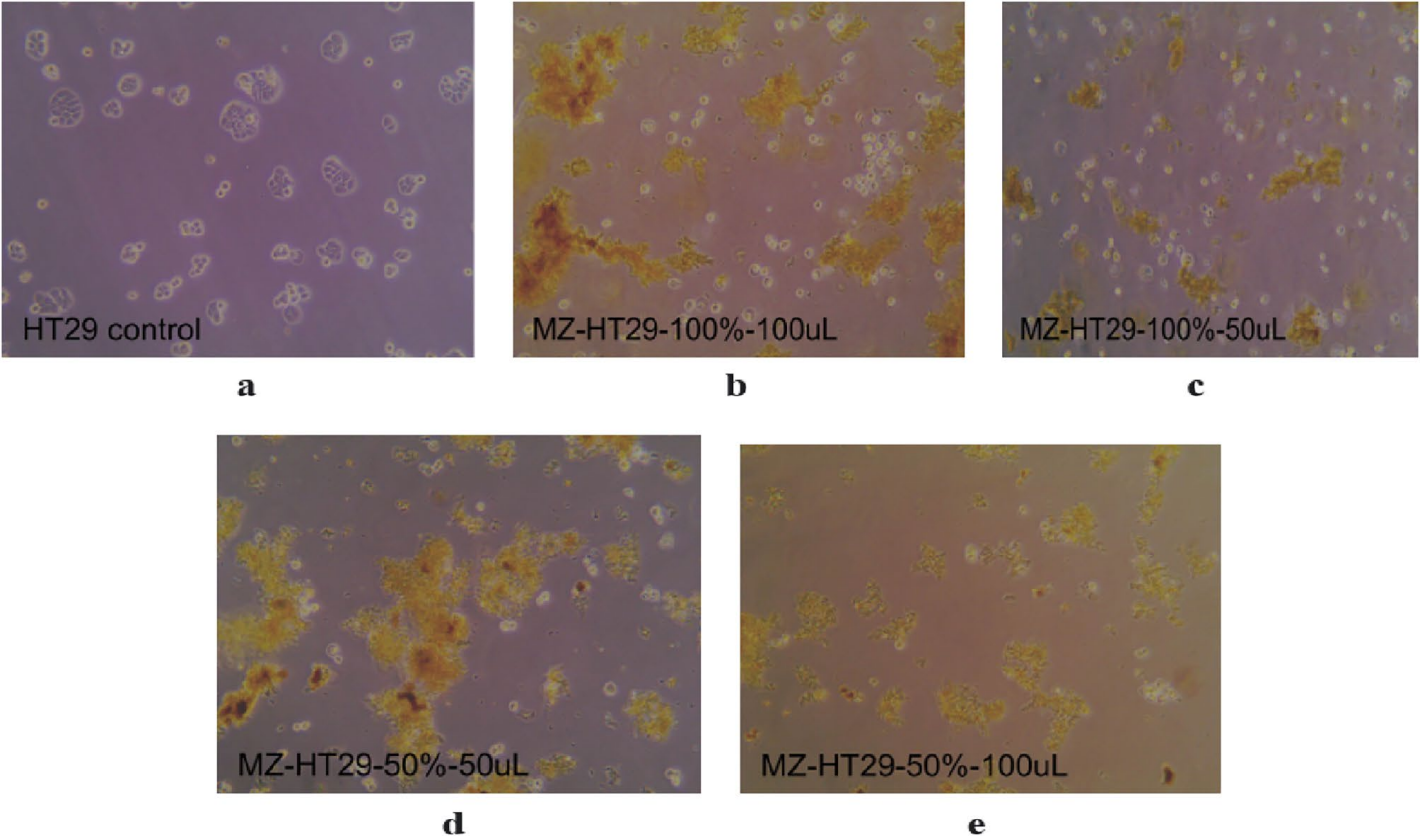
The effect of *C. persicum* on HT29: (**a**) The control samples of the HT29 cell lines 48 h after culturing with DMSO (**b**). HT29 cell lines 48 h after culturing with 100 μL of 100% extract (**c**). HT29 cell lines 48 h after culturing with 50 μL of 100% extract (**d**). HT29 cell lines 48 h after culturing with 50 μL of 50% extract (**e**). HT29 cell lines 48 h after culturing with 100 μL of 50% extract.

The study revealed that the *C. persicum* extracts exhibited significant anti-HT29 activity, specifically against the HT29 colon cancer cell line. When comparing the activity against HT29, the 100% ethanolic extract of *C. persicum* showed higher activity compared to the 50% ethanolic extract, as depicted in Fig. 5b–e of the study.

Additionally, when comparing the activity of *C. persicum* extracts against the two cancer cell lines (MCF7 and HT29), it was observed that the extract had a greater effect on HT29 than on MCF7, even when the same amount of extract was used. This suggests that the anti-cancer activity of *C. persicum* may vary depending on the specific cancer cell line, and HT29 cells may be more sensitive to the effects of the extract compared to MCF7 cells. The results from Fig. 5a–e provide evidence of this differential response between the two cell lines.

## Conclusion

*Cyclamen persicum*, a plant with a longstanding history of medicinal use, possesses nutraceutical, pharmaceutical, and medicinal properties. This can be attributed to it is rich content of polyphenolic substances and flavonoids, which are important secondary metabolites for their diverse biological activities. HPLC analysis of the *C. persicum* extracts revealed the presence of various flavonoids and phenolic compounds. These include rutin, sinapic acid, chlorogenic acid, kaempferol, quercetin, trans-cinnamic acid, and p-coumaric acid. The presence of these flavonoids and polyphenolic substances contributes to the antioxidant activity and other biological activities exhibited by *C. persicum*. The plant extract also demonstrates mild inhibitory activity against both Gram-positive and Gram-negative bacterial strains. This suggests its potential as a natural antibacterial agent. Furthermore, *C. persicum* exhibits notable anti-cancer properties in colon (HT29) and breast (MCF7) cancer cell lines. This indicates its potential as a natural source of anti-cancer compounds. Overall, the diverse biological activities, including antioxidant, antibacterial, and anti-cancer properties, of *C. persicum* can be attributed to its rich composition of flavonoids and polyphenolic substances. These findings support the traditional medicinal use of *C. persicum* and highlight its potential for further exploration as a source of therapeutic agents.

## Data Availability

All data generated or analyzed during this study are included in this published article.
