# Author Correction: Anticancer, antioxidant, and antibacterial activity of chemically fingerprinted extract from *Cyclamen persicum* Mill.

**DOI:** 10.1038/s41598-024-65528-w

**Published:** 2024-06-25

**Authors:** Fuad Al‑Rimawi, Mahmoud Khalid, Zaidoun Salah, Malak A. A. Zawahreh, Sulaiman Mohammed Alnasser, Shifaa O. Alshammari, Fadel Wedian, Shaik Karimulla, Abdulrahman Almutairi, Fadi Ibrahim B. Alanazi, Hamad Owijan Alanazi, Ghassab M. Al‑Mazaideh, Hiba‑Allah Nafidi, Ahmad Mohammad Salamatullah, Amare Bitew Mekonnen, Mohammed Bourhia

**Affiliations:** 1 Chemistry Department, Faculty of Science and Technology, Al-Quds University, P.O. Box 2002, Jerusalem, Palestine; 2 Molecular Genetics and Genetic Toxicology Program, Arab American University, Ramallah, Palestine; 3 Department of Pharmacology and Toxicology, College of Pharmacy, Qassim University, 51452 Qassim, Saudi Arabia; 4 Department of Biology, College of Science, University of Hafr Al Batin, P.O. Box 1803, Hafr Al Batin, Saudi Arabia; 5 Department of Chemistry, Faculty of Science, Yarmouk University, P.O. Box 560, Irbid, 22163 Jordan; 6 Department of Pharmacy Practice, University of Hafr Al Batin, Hafr Al Batin, Saudi Arabia; 7 Disaster and Emergency Services Department, Health Affairs Directorate, Hafr Al Batin, Kingdom of Saudi Arabia; 8 Department of Pharmaceutical Chemistry, College of Pharmacy, University of Hafr Al Batin, P.O. Box 1803, 31991 Hafr Al Batin, Saudi Arabia; 9 Department of Food Science, Faculty of Agricultural and Food Sciences, Laval University, Quebec City, QC 2325G1V 0A6 Canada; 10 Department of Food Science and Nutrition, College of Food and Agricultural Sciences, King Saud University, 11, P.O. Box 2460, 11451 Riyadh, Saudi Arabia; 11 Department of Biology, Bahir Dar University, P.O. Box 79, Bahir Dar, Ethiopia; 12 Laboratory of Biotechnology and Natural Resources Valorization, Faculty of Sciences, Ibn Zohr University, 80060 Agadir, Morocco; 13 Biology Program, Natural Sciences Division, Al-Quds Bard College, Al-Quds University, Al-Quds, Palestine

Correction to: *Scientific Reports* 10.1038/s41598-024-58332-z, published online 11 April 2024

The original version of this Article contained an error in the Abstract.

“The experimental results revealed weak antibacterial activity of *C. persicum* extract against both gram negative (*E. coli*) and gram positive (*Streptococcus aureus* and *S. aureus*) bacterial strains, with the zones of inhibition found to be less than 8 mm.”

now reads:

“The experimental results demonstrated limited antibacterial activity of *C. persicum* extract against both gram-negative (*E. coli*) and gram-positive (*Streptococcus* and *S. aureus*) bacterial strains, with the observed zones of inhibition measuring less than 8 mm.”

The original Article has been corrected.

